# Changes in lung function and exercise capacity are strong predictors of mortality in patients with IPF receiving antifibrotic therapy

**DOI:** 10.3389/fmed.2025.1679011

**Published:** 2025-10-07

**Authors:** Ju Hyun Oh, Moo Suk Park, Man Pyo Chung, Sung Hwan Jeong, Jin Woo Song, Sun Mi Choi, Yong Hyun Kim, Sung Woo Park, Yangin Jegal, Hee-Young Yoon, Won-Il Choi, Jung-Wan Yoo, Hyun-kyung Lee, Sei-Hoon Yang, Eun-Joo Lee, Hye Sook Choi, Hyung Koo Kang, Jong Sun Park, Jae Ha Lee

**Affiliations:** ^1^Department of Pulmonary and Critical Care Medicine, Ajou University Medical Center, Ajou University School of Medicine, Suwon, Republic of Korea; ^2^Division of Pulmonary and Critical Care Medicine, Department of Internal Medicine, Severance Hospital, Yonsei University College of Medicine, Seoul, Republic of Korea; ^3^Division of Pulmonary and Critical Care Medicine, Department of Medicine, Samsung Medical Center, Sungkyunkwan University School of Medicine, Seoul, Republic of Korea; ^4^Department of Allergy, Pulmonology and Critical Care Medicine, Gil Medical Center, Gachon University, Incheon, Republic of Korea; ^5^Division of Pulmonary and Critical Care Medicine, Asan Medical Center, University of Ulsan College of Medicine, Seoul, Republic of Korea; ^6^Division of Pulmonary and Critical Care Medicine, Department of Internal Medicine, Seoul National University Hospital, Seoul National University College of Medicine, Seoul, Republic of Korea; ^7^Division of Pulmonary and Critical Care Medicine, Department of Internal Medicine, Bucheon St. Mary’s Hospital, Catholic University of Korea School of Medicine, Bucheon-si, Republic of Korea; ^8^Division of Allergy and Respiratory Medicine, Department of Internal Medicine, Soonchunhyang University Bucheon Hospital, Bucheon-si, Republic of Korea; ^9^Division of Pulmonary Medicine, Department of Internal Medicine, Ulsan University Hospital, University of Ulsan College of Medicine, Ulsan, Republic of Korea; ^10^Division of Pulmonary and Critical Care Medicine, Department of Internal Medicine, Soonchunhyang University Seoul Hospital, Soonchunhyang University College of Medicine, Seoul, Republic of Korea; ^11^Division of Pulmonary and Critical Care Medicine, Department of Internal Medicine, Myongji Hospital, Goyang, Republic of Korea; ^12^Department of Internal Medicine, Gyeongsang National University Hospital, Gyeongsang National University College of Medicine, Jinju, Republic of Korea; ^13^Division of Pulmonary, Allergy, and Critical Care Medicine, Department of Internal Medicine, Inje University Busan Paik Hospital, Busan, Republic of Korea; ^14^Division of Pulmonary, Department of Internal Medicine, College of Medicine, Wonkwang University, Iksan, Republic of Korea; ^15^Division of Respiratory and Critical Care Medicine, Department of Internal Medicine, Korea University Anam Hospital, Korea University College of Medicine, Seoul, Republic of Korea; ^16^Department of Pulmonary and Critical Care Medicine, Kyung Hee University Medical Center, School of Medicine, Kyung Hee University, Seoul, Republic of Korea; ^17^Division of Pulmonology and Critical Care Medicine, Department of Internal Medicine, Inje University Ilsan Paik Hospital, Inje University College of Medicine, Busan, Republic of Korea; ^18^Division of Pulmonary and Critical Care Medicine, Department of Internal Medicine, Seoul National University Bundang Hospital, Seoul National University College of Medicine, Seongnam, Republic of Korea; ^19^Division of Pulmonology and Critical Care Medicine, Department of Internal Medicine, Inje University Haeundae Paik Hospital, Inje University College of Medicine, Busan, Republic of Korea

**Keywords:** interstitial lung disease, fibrosis, prognosis, mortality, lung function

## Abstract

**Background:**

Idiopathic pulmonary fibrosis (IPF) is a chronic fibrosing interstitial pneumonia with poor prognosis. This study evaluated whether monitoring changes in lung function and exercise capacity during antifibrotic therapy offers superior prognostic value compared with baseline clinical parameters in IPF.

**Methods:**

We retrospectively analyzed patients with IPF enrolled with the Korean IPF cohort registry between June 2016 and August 2021. Prognostic factors for mortality were assessed using Cox proportional hazards models and receiver operating characteristic (ROC) curve analysis.

**Results:**

Among 1,229 patients (mean age 68.3 years; 82.8% male), 88.0% received antifibrotic therapy. During a median follow-up of 41.0 months, 37.9% of the treated patients died. Multivariable Cox analysis revealed that a decline in forced vital capacity (FVC) at 12 months, lower baseline diffusing capacity of the lungs for carbon monoxide (DLco), a decline in DLco at 12 months, and a reduction in the 6-min walk distance at 6 months, were independent risk factors for mortality in IPF patients receiving antifibrotic therapy. In the ROC curve analysis, the change in FVC at 12 months showed the highest predictive accuracy for mortality (area under the curve = 0.676; *p* < 0.001). Kaplan–Meier analysis demonstrated significantly poorer survival in patients with ≥5.8% decline in FVC and ≥11.5% decline in DLco over 12 months (*p* < 0.001 and *p* = 0.001, respectively).

**Conclusion:**

Longitudinal changes in lung function and exercise capacity as indicators of response to antifibrotic therapy may serve as potential surrogate markers of mortality in patients with IPF.

## Introduction

Idiopathic pulmonary fibrosis (IPF) is a chronic, progressive, fibrosing interstitial lung disease of unknown cause characterized by a poor prognosis (1, 2). The clinical course of IPF is heterogeneous, ranging from slowly progressive disease to rapid deterioration, and patient median survival has been reported to be approximately 3–5 years post-diagnosis (1, 2).

Several factors associated with increased mortality in IPF patients have been identified, including older age, lower body mass index (BMI), male sex, and presence of comorbidities (3–5). Furthermore, physiological measures, including baseline pulmonary function—particularly forced vital capacity (FVC) and diffusing capacity of the lung for carbon monoxide (DLco)—and exercise capacity as assessed by the 6-min walk test (6MWT), have been widely recognized as key prognostic indicators (6–8). These static parameters at the time of diagnosis have been commonly used for staging disease severity and estimating survival, such as in the Gender-Age-Physiology (GAP) model (9–11). However, prognostic models based on baseline status have limitations, because they do not reflect treatment responses or disease progression. Longitudinal changes in lung function and exercise capacity are reportedly associated with patient outcomes (12–15). Notably, Salisbury et al. found that GAP stage did not predict the rate of lung function decline, whereas a decline of ≥10% in FVC or DLco over 6 months independently predicted death or lung transplantation in IPF patients (12).

Moreover, the introduction of antifibrotic therapies, such as pirfenidone and nintedanib, has led to a paradigm shift in the management of patients with IPF, prompting increased interest in dynamic prognostic indicators that reflect treatment responses and disease activity over time (16, 17). In this context, longitudinal changes in lung function and exercise capacity, particularly in patients receiving antifibrotic therapy, may better reflect responses to treatment and provide superior prognostic value compared to baseline measurements alone (18). Harari et al. demonstrated that, in patients with IPF treated with pirfenidone, the GAP index had limited short-term prognostic accuracy for mortality (Hosmer-Lemeshow *p* = 0.014 and 0.019 at 1 and 2 years, respectively), suggesting that predictions based on baseline information could be insufficient in the antifibrotic era (18).

In this study, we aimed to investigate whether longitudinal changes in lung function and exercise capacity could accurately predict mortality in patients with IPF, particularly in those receiving antifibrotic therapy. By examining dynamic physiological parameters in a large multicenter Korean IPF cohort (KICO) registry, we sought to identify reliable surrogate markers to better inform clinical decision-making for individualized patient management.

## Materials and methods

### Study population

This retrospective study included patients with IPF registered in the KICO registry between June 2016 and August 2021 (NCT04160715). The KICO registry is a multi-center database of 23 medical institutions across South Korea. IPF was diagnosed through multidisciplinary discussions based on the criteria of the American Thoracic Society/European Respiratory Society guidelines (19, 20). A total of 2,367 patients with IPF were initially screened. Of these, 252 were excluded for missing baseline, follow-up, or treatment data, and 886 for receiving other treatments such as corticosteroids or immunosuppressants. Thus, 1,229 patients were included in the final analysis. This study was approved by the Institutional Review Board of Haeundae Paik Hospital (approval no. 2025–05-031). The requirement for written informed consent was waived due to the retrospective and de-identified nature of this study.

### Clinical data

Clinical information, including age, sex, body mass index (BMI), smoking history, comorbidities, antifibrotic treatment, including pirfenidone and nintendanib, home oxygen use, and survival status, were collected from the KICO web-based registry data which applies standardized quality control procedures. Pulmonary function test (PFT) results, including FVC and DLco, and outcomes from the 6MWT, including baseline SpO₂, nadir SpO₂, and 6-min walk distance (6MWD), were obtained from the KICO web-based registry at the time of diagnosis, at 6 months, and at 12 months follow-up. The GAP score at diagnosis was calculated based on four clinical variables: sex, age, FVC, and DLco; patients were classified into three GAP stages according to their total score: stage I (0–3 points), stage II (4–5 points), and stage III (6–8 points), as previously described (10).

### Statistical analysis

Continuous and categorical variables are expressed as means ± standard deviation and percentages, respectively. Comparisons between groups were performed using Student’s *t*-test or Mann–Whitney *U*-test for continuous variables and the Chi-squared test or Fisher’s exact test for categorical variables, as appropriate. Lung function changes were calculated as the difference in % predicted FVC or DLco from baseline, as follows: ΔFVC or ΔDLco at 12 months = FVC or DLco (% predicted) at 12 months – FVC or DLco (% predicted) at baseline. Changes in 6MWD were also calculated as: Δ6MWD (meters) at 12 months = 6MWD (meters) at 12 months – 6MWD (meters) at baseline. Univariate and multivariate Cox proportional hazards regression analyses were performed to identify prognostic factors independently related to overall survival. Variables with a *p*-value of <0.1 in the univariate analysis were included in the multivariable analysis using backward elimination. To assess the predictive value of decline in lung function and exercise capacity for mortality in patients with IPF, receiver operating characteristic (ROC) curve analysis was conducted, and the optimal cutoff value was calculated using the Youden index method (21). Survival was evaluated using Kaplan–Meier survival analysis and the log-rank test. All statistical analyses were performed using SPSS v.29.0 statistical software (IBM Corp. Released 2023. IBM SPSS Statistics for Windows, v.29.0.2.0 Armonk, NY: IBM Corp) and R statistical software.^1^ All *p*-values were two-sided, and p-values <0.05 were considered statistically significant.

## Results

### Baseline characteristics of IPF patients

Among the 1,229 patients with IPF, the mean age was 68.3 years, and 82.8% were male (Table 1). A total of 88.0% (1,085/1,229) received antifibrotic therapy with a median treatment duration of 15.3 months. A total of 144 patients were not treated with antifibrotics. Compared with those receiving antifibrotic therapy, patients in the no-treatment group were older and had a higher prevalence of lung cancer, lower BMI, and poorer exercise capacity, despite having relatively preserved pulmonary function. There was no significant difference in home oxygen use at baseline; however, it was significantly more common during follow-up in the antifibrotic treatment group (20.5% vs. 6.3%; *p* < 0.001).

**Table 1:** Baseline characteristics of patients with IPF.

Variable	Overall	Antifibrotics	No treatment	*p*-value
All patients	1,229	1,085	144	
Male	1,018 (82.8)	902 (83.1)	116 (80.6)	0.441
Age, year	68.28 ± 8.14	68.04 ± 8.13	70.12 ± 8.05	0.004
BMI, Kg/m^2^	24.23 ± 3.11	24.35 ± 3.09	23.30 ± 3.11	<0.001
Ever smoker	780 (65.0)	690 (65.3)	90 (62.9)	
Lung cancer history	123 (10.0)	99 (9.1)	24 (16.7)	0.005
Pulmonary function test				
FVC, % predicted				
baseline	75.55 ± 16.02	74.96 ± 15.77	80.16 ± 17.26	<0.001
Δ at 6 months	−0.78 ± 8.10	−0.86 ± 8.13	0.04 ± 7.81	0.350
Δ at 1 year	−1.95 ± 8.95	−1.91 ± 8.86	−2.43 ± 9.99	0.637
DLco, % predicted				
baseline	62.36 ± 19.57	61.94 ± 19.41	65.83 ± 20.61	0.042
Δ at 6 months	−1.47 ± 11.77	−1.60 ± 12.05	−0.18 ± 8.48	0.342
Δ at 1 year	−3.20 ± 13.12	−3.40 ± 13.07	−0.79 ± 13.58	0.132
Six-minute walk test				
Distance	416.54 ± 107.53	419.59 ± 106.04	382.93 ± 118.39	0.006
Baseline SpO_2_	95.89 ± 5.87	95.88 ± 5.93	96.50 ± 1.73	0.835
Nadir SpO_2_	90.23 ± 7.16	90.27 ± 7.00	89.78 ± 8.84	0.647
GAP stage				
Stage I	641 (57.0)	565 (56.6)	76 (59.8)	0.014
Stage II	388 (34.5)	355 (35.6)	33 (26.0)	
Stage III	96 (8.5)	78 (7.8)	18 (14.2)	
Home oxygen use				
At baseline	22 (1.8)	21 (1.9)	1 (0.7)	0.502
During follow up	224 (18.8)	215 (20.5)	9 (6.3)	<0.001
Lung transplantation	24 (2.0)	23 (2.1)	1 (0.7)	0.347

Data are presented as mean ± SD or numbers (%), unless otherwise indicated. BMI, body mass index; FVC, forced vital capacity; DLco, diffusing capacity of lungs for carbon monoxide; SpO_2_, percutaneous oxygen saturation; GAP, sex-age-physiology.

### Longitudinal changes in lung function and exercise capacity

**Figure 1:**
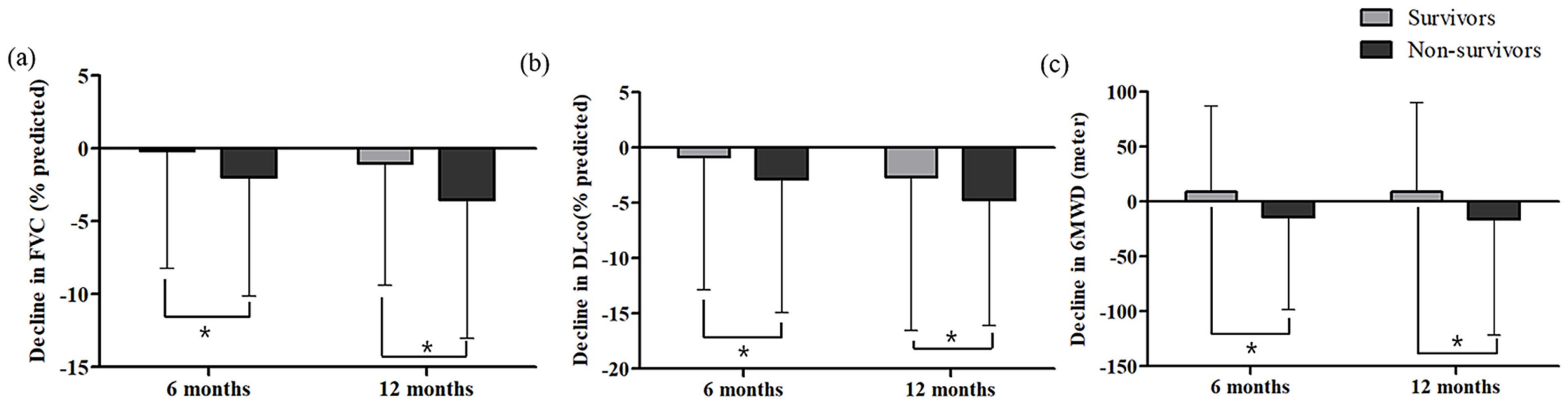
Changes in **(a)** FVC, **(b)** DLco and **(c)** 6MWD at 6 and 12 months according to survival status in patients with IPF receiving antifibrotics. FVC, forced vital capacity; DLco, diffusing capacity of lungs for carbon monoxide; 6MWD, six-minute walk distance. *Asterisks indicate statistically significant differences (*p* < 0.05) between non-survivors and survivors in the change from baseline to 6 or 12 months in FVC, DLco, and 6MWD.

During follow-up (median, 41.0 months; interquartile range [IQR], 22.0–62.0), 37.9% (411/1,085) and 51.4% (74/144) of patients in the antifibrotic treatment and no-treatment groups, respectively, had died. In both groups, baseline FVC, DLco, and 6MWD were lower in non-survivors than in survivors (Supplementary Table S1). Over the follow-up period, in the antifibrotic treatment group, FVC declined more substantially in the non-survivors compared to the survivors at both 6 months (−2.01 ± 8.14% vs. –0.19 ± 8.05%, *p* = 0.003) and 1 year (−3.54 ± 9.51% vs. –1.04 ± 8.37%, *p* < 0.001; Figure 1a). Similarly, DLco declined more markedly in the non-survivors at 6 months (−2.84 ± 12.10% vs. –0.89 ± 11.97%, *p* = 0.043) and at 1 year (−4.57 ± 11.36% vs. –2.70 ± 13.84%, *p* = 0.046) in the antifibrotic treatment group (Figure 1b). Additionally, the non-survivors exhibited a significantly greater decline in 6MWD at both 6 months (−14.58 ± 73.66 meters vs. 8.99 ± 77.83 meters, *p* = 0.013) and 1 year (−15.89 ± 106.05 meters vs. 8.74 ± 81.51 meters, *p* = 0.011) when compared to survivors in the antifibrotic treatment cohort (Figure 1c). In contrast, in the no-treatment group, no statistically significant differences existed in longitudinal changes in lung function parameters and 6MWD between non-survivors and survivors over the 1-year follow-up period (Supplementary Figure S1).

### Risk factors for mortality

In univariate Cox proportional hazards analysis, older age, lower BMI, and home oxygen use were significantly associated with increased mortality in patients with IPF receiving antifibrotics (Table 2). Among lung function parameters, lower baseline FVC and DLco, as well as greater declines in FVC at 6 and 12 months, and in DLco at 12 months, were significantly associated with increased mortality risk (Table 2). In the 6MWT, lower nadir SpO₂ and greater reductions in 6MWD at both 6 and 12 months were significantly associated with mortality in univariate analysis. In multivariate Cox analysis, greater FVC decline at 12 months (hazard ratio [HR] = 0.95; 95% confidence interval [CI], 0.91–0.99; *p* = 0.028), lower baseline DLco (HR = 0.94; 95% CI, 0.92–0.96; *p* < 0.001), greater DLco decline at 12 months (HR = 0.96; 95% CI, 0.92–1.00; *p* = 0.035), and a greater reduction in 6MWD at 6 months (HR = 0.99; 95% CI, 0.99–1.00; *p* = 0.039) remained significant risk factors for mortality (Table 2). In patients with IPF not receiving antifibrotics, a greater decline in DLco at 12 months (HR = 0.92; 95% CI, 0.87–0.97; *p* = 0.001) and a shorter 6MWD at baseline (HR = 0.99; 95% CI, 0.98–1.00; *p* = 0.005) were identified as independent prognostic factors for mortality in the multivariate Cox proportional hazards model (Supplementary Table S2).

**Table 2:** Prognostic factors for mortality in patients with IPF receiving antifibrotics in the cox proportional hazards model.

Variable	Univariate analysis		Multivariate analysis	
	HR (95% CI)	*p*-value	HR (95% CI)	*p*-value
Age	1.06 (1.04–1.07)	<0.001		
Male	1.11 (0.86–1.44)	0.416		
BMI	0.93 (0.90–0.96)	<0.001		
Ever smokers	0.94 (0.75–1.17)	0.573		
Lung cancer	1.21 (0.88–1.65)	0.242		
Home oxygen use^†^	1.76 (1.42–2.17)	<0.001		
Pulmonary function				
FVC				
Baseline	0.97 (0.96–0.97)	<0.001		
Δ at 6 months	0.97 (0.96–0.99)	<0.001		
Δ at 1 year	0.97 (0.95–0.98)	<0.001	0.95 (0.91–0.99)	0.028
DLCO				
Baseline	0.96 (0.96–0.97)	<0.001	0.94 (0.92–0.96)	<0.001
Δ at 6 months	0.99 (0.98–1.00)	0.087		
Δ at 1 year	0.99 (0.98–1.00)	0.032	0.96 (0.92–1.00)	0.035
Six-minute walk test				
Baseline SpO_2_	0.99 (0.96–1.02)	0.547		
Nadir SpO_2_	0.96 (0.95–0.96)	<0.001		
Distance				
Baseline	1.00 (1.00–1.00)	<0.001		
Δ at 6 months	1.00 (1.00–1.00)	0.038	0.99 (0.99–1.00)	0.039
Δ at 1 year	1.00 (0.99–1.00)	0.002		
GAP stage				
Stage I	ref			
Stage II	2.96 (2.37–3.69)	<0.001		
Stage III	4.18 (3.00–5.82)	<0.001		

HR, hazards ratio; CI, confidence interval; BMI, body mass index; FVC, forced vital capacity; DLco, diffusing capacity of lungs for carbon monoxide; SpO_2_, percutaneous oxygen saturation; GAP, sex-age physiology. ^†^Home oxygen use during the follow-up period.

**Figure 2:**
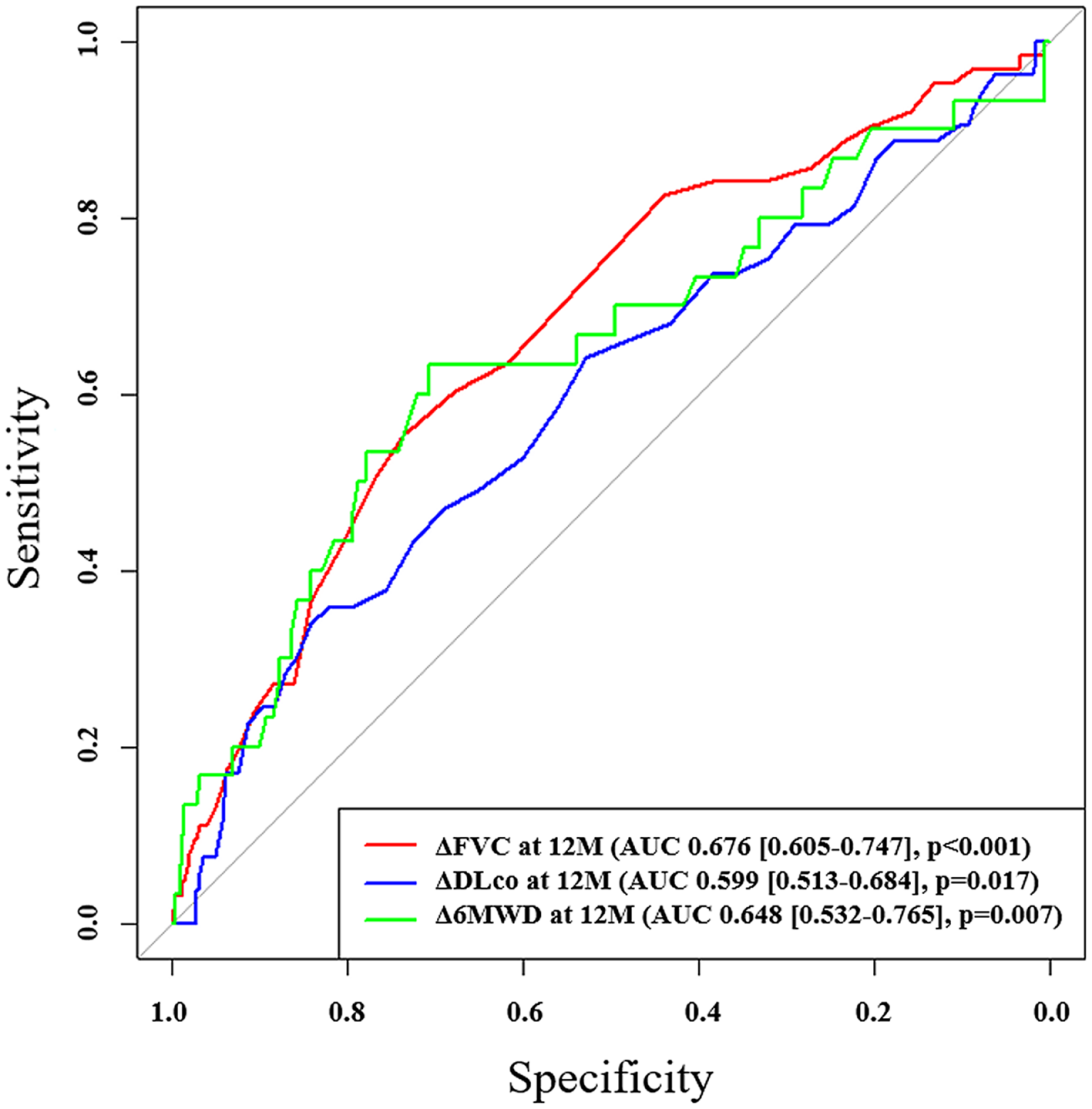
ROC analysis of changes in pulmonary function and exercise capacity for predicting mortality in patients with IPF receiving antifibrotics. ROC, receiver operating characteristic; AUC, area under the curve; FVC, forced vital capacity; DLco, diffusing capacity of lungs for carbon monoxide; 6MWD, six-minute walk distance.

**Figure 3:**
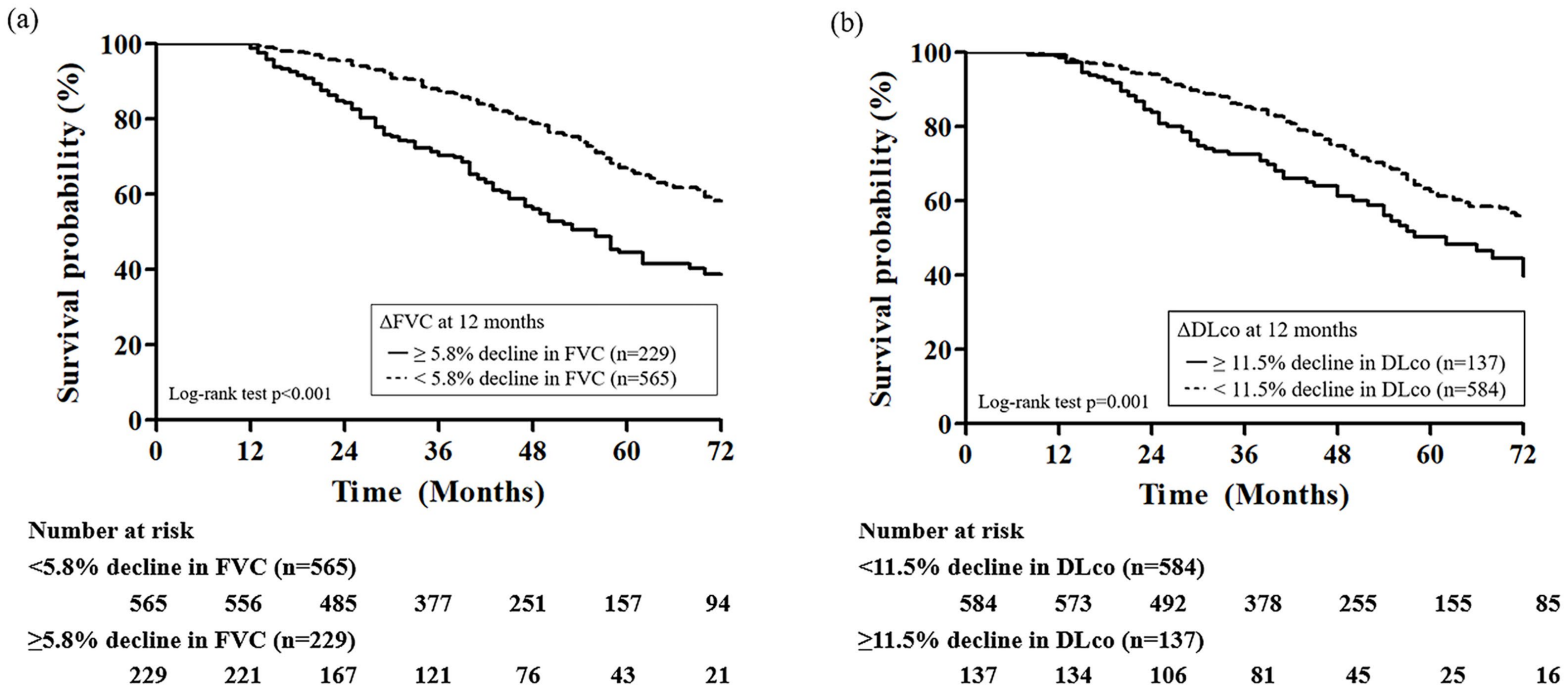
Kaplan–Meier survival curves according to 12-month changes in **(a)** FVC and **(b)** DLco in patients with IPF receiving antifibrotics. FVC, forced vital capacity; DLco, diffusing capacity of lungs for carbon monoxide.

In ROC analysis, a decline in FVC during the first 12 months demonstrated the highest predictive accuracy [area under the curve (AUC) = 0.676; 95% CI, 0.605–0.747; *p* < 0.001; cutoff value, −5.8%] and mortality during the subsequent 1-year follow-up period, whereas a decline in DLco over the same period also showed a significant but lower predictive value (AUC = 0.599; 95% CI, 0.513–0.684; *p* = 0.017; cutoff value, −11.5%; Figure 2). Additionally, the 12-month change in 6MWD was significantly predictive of mortality (AUC = 0.648; 95% CI, 0.532–0.765; *p* = 0.007) in IPF patients receiving antifibrotics. In patients with IPF not receiving antifibrotic therapy, only the decline in DLco at 12 months demonstrated significant predictive performance for mortality (AUC = 0.869; *p* = 0.003), whereas FVC and 6MWD changes did not (Supplementary Table S3). Kaplan–Meier analysis showed that, among IPF patients receiving antifibrotics, those with an FVC decline of ≥5.8% at 12 months (*n* = 229) had significantly poorer overall survival compared to those with a decline <5.8% (*n* = 565) (log-rank *p* < 0.001; Figure 3a). Similarly, patients with a DLco decline of ≥11.5% over 12 months (*n* = 137) exhibited significantly poorer survival than those with a smaller decline (*n* = 584) (*p* = 0.001; Figure 3b).

## Discussion

In this study, we demonstrated that longitudinal declines in FVC and DLco over 12 months, as well as a decline in 6MWD at 6 months, which reflects treatment responses, were more significantly associated with mortality than baseline lung function and exercise capacity in patients with IPF receiving antifibrotic therapy. These findings underscore the prognostic importance of dynamic physiological changes over static baseline measurements in risk stratification and support individualized treatment strategies for patients receiving antifibrotic therapy.

In the present study, greater declines in FVC and DLco over 12 months were independently associated with increased mortality in IPF patients receiving antifibrotic therapy. Notably, our study identified a ≥ 5.8% decline in FVC and a ≥ 11.5% decline in DLco over 12 months as optimal cutoffs predictive of mortality. These values are comparable to the thresholds used to define physiological progression in the recent clinical definition of progressive pulmonary fibrosis, which include a 5% absolute decline in FVC or a 10% absolute decline in DLco (1). Therefore, in IPF patients receiving antifibrotic therapy, those who exhibit declines beyond these cutoff values may be classified as a high-risk group with poor prognosis, for whom closer monitoring and additional therapeutic interventions are required. The finding that progressive declines in FVC and DLco strongly predict adverse clinical outcomes in patients with IPF is consistent with previous prognostic evidence (13, 14). In a report by Collard et al., which included 51 IPF patients, a decline of ≥10% in FVC over 12 months was associated with a nearly fourfold increase in mortality risk (HR = 3.95; 95% CI, 1.62–9.62), and a ≥ 15% decline in DLco was associated with a 2.6-fold increase in mortality risk (HR = 2.61; 95% CI, 1.04–6.55) (13). Furthermore, a recent study from the IPF-PRO Registry cohort demonstrated that even small absolute declines in FVC (≥ 2%; adjusted HR = 1.80; 95% CI, 1.42–2.28) and DLco (≥ 2%; adjusted HR = 2.04; 95% CI, 1.34–3.10) over 12 months are significantly associated with increased risks of mortality or lung transplantation (14). However, these previous studies evaluated the association between lung function decline and outcomes in the entire IPF population without separately analyzing effects in patients receiving antifibrotic therapy, as in the present study.

Additionally, a greater reduction in the 6MWD at 6 months was independently associated with an increased risk of death in IPF patients who were receiving antifibrotics. Previous investigations have similarly identified early deterioration in 6MWD as a robust predictor of subsequent disease progression and mortality in IPF (15, 22, 23). A study by du Bois et al., including the INSPIRE trial cohort, demonstrated that a 50 m decline in 6MWD over 24 weeks is associated with a nearly three-fold increased risk of mortality in the following year (HR = 2.73; 95% CI, 1.60–4.66) and enhanced prognostic accuracy when incorporated into a model that includes age, respiratory hospitalization, FVC % predicted, and 24-week change in FVC % predicted (22). However, previous studies exploring the association between mortality and changes in 6MWD relied on stratified threshold-based analyses (< 25 m as a reference group, 26–50 m, or > 50 m), which, although informative, may exaggerate effect sizes in critically ill subgroups and limit the generalizability of the findings (15, 22). Notably, in our study, IPF patients receiving antifibrotics who survived exhibited an improvement in 6MWD over time, while even in the non-survivor group, the average 6-month decline was <15 meters. These findings suggest that subtle functional changes, even those within a minimal range, may have prognostic significance and should be closely monitored during follow-up.

This study had certain limitations. First, because the study cohort consisted exclusively of Korean IPF patients, the generalizability of our findings to other regional, ethnic, and international populations may be limited. However, baseline characteristics were comparable to those reported in other IPF cohorts (14, 15). Second, the use of all-cause mortality as the outcome, rather than respiratory-related death, may have introduced confounding factors unrelated to IPF progression. Third, detailed information concerning antifibrotic dose and dose adjustment was not available, precluding analysis of dose–response relationships and their potential impact on clinical outcomes. Fourth, we analyzed lung function and exercise capacity data only up to 1 year post-diagnosis, which limits the ability to assess long-term physiological changes and treatment responses. Fifth, the relatively modest AUC values likely reflect the limitations of single-marker analyses, as prognosis in IPF is multifactorial; thus, integrated multivariable models are needed for improved predictive performance in future studies. Despite these limitations, the strengths of our study include its large sample size, multicenter design encompassing diverse clinical settings, and standardized data collection methodology, all of which enhance the generalizability of our findings. In addition, because most patients received antifibrotic therapy, our results provide clinically relevant insights into real-world outcomes among patients treated with antifibrotics.

In conclusion, our findings suggest that longitudinal declines in FVC, DLco, and 6MWD, which reflect responses to antifibrotic therapy, serve as more important surrogate markers of mortality than baseline clinical status in IPF patients receiving antifibrotic treatment. Therefore, serial assessments of pulmonary function and exercise capacity should be incorporated into clinical practice to enable early identification of high-risk patients. For these patients, more proactive treatment strategies, including dose escalation of antifibrotic agents, off-label combination therapy, and participation in clinical trials, should be considered.

## Data Availability

The raw data supporting the conclusions of this article will be made available by the authors, without undue reservation.

## Footnotes

^1^v.4.2.1; R Foundation, Vienna, Austria; http://www.r-project.org/
