# Supplementary material for: Changes in lung function and exercise capacity are strong predictors of mortality in patients with IPF receiving antifibrotic therapy
Source: Front Med (Lausanne). 2025 Oct 7;12:1679011. doi: 10.3389/fmed.2025.1679011 (PMC12537395; doi:10.3389/fmed.2025.1679011)
Supplement: Supplementary file 1 [file Data_Sheet_1.pdf]

Supplementary Material

Table S1. Comparison of baseline characteristics between survivors and non-survivors

	**Antifibrotics (n = 1,085)**			**No treatment (n = 144)**		
**Characteristics**	Non-survivor	Survivor	p- value	Non-survivor	Survivor	p-value
Number	411 (37.9)	674 (62.1)		74 (51.4)	70 (48.6)	
Male	342(83.2)	560(83.1)	0.957	62(83.8)	54(77.1)	0.314
Age, year	69.13±7.85	67.38±8.23	0.001	72.08±6.77	68.04±8.80	0.003
BMI, Kg/m^2	23.83±3.17	24.67±3.00	<0.001	22.75±3.51	23.88±2.53	0.029
Ever smoker	270(67.8)	420(63.7)	0.001	50(68.5)	40(57.1)	0.318
Lung cancer	44 (10.7)	55 (8.2)	0.158	15 (20.3)	9 (12.9)	0.233
PFT						
FVC, % pred	70.33±15.92	77.74±15.02	<0.001	73.95±17.43	86.27±14.83	<0.001
DLco, % pred	54.95±18.12	66.11±18.96	<0.001	57.36±19.54	73.61±18.51	<0.001
6MWT						
Distance	396.29±111.94	435.23±98.98	<0.001	346.18±111.38	415.81±116.10	<0.001
Nadir SpO_2	88.14±6.95	91.72±6.66	<0.001	86.68±10.78	92.55±5.46	0.012
GAP stage						
Stage I	159(42.1)	406(65.5)	<0.001	25(39.7)	51(79.7)	<0.001
Stage II	173(45.8)	182(29.4)		23(36.5)	10(15.6)	
Stage III	46(12.2)	32(5.2)		15(23.8)	3(4.7)	
Home oxygen use†	131(33.1)	84(12.9)	<0.001	5(6.8)	4(5.7)	0.535

Data are presented as means ± SD or number (%), unless otherwise indicated. BMI, body mass index; PFT, pulmonary function test; FVC, forced vital capacity; DLco, diffusing capacity of the lung for carbon monoxide; 6 MWT, six-minute walk test; SpO_2, percutaneous oxygen saturation; GAP, sex–age–physiology.
†Home oxygen use during the follow-up period.

Table S2. Prognostic factors for mortality in patients with IPF not receiving antifibrotic therapy in Cox proportional hazards model

Variable	Univariate Analysis		Multivariate Analysis	
	HR (95% CI)	p- value	HR (95% CI)	p- value
Age	1.06 (1.03-1.09)	<0.001		
Male	1.20 (0.64-2.23)	0.570		
BMI	0.91 (0.83-0.98)	0.018		
Ever smokers	1.44 (0.77-2.67)	0.253		
Lung cancer	1.63 (0.92-2.88)	0.110		
Home oxygen use†	1.76 (0.70-4.39)	0.227		
Pulmonary function				
FVC				
baseline	0.96 (0.95-0.98)	<0.001		
Δ at 6 months	0.98 (0.94-1.03)	0.493		
Δ at 1 year	0.96 (0.92-0.99)	0.019		
DLCO				
baseline	0.96 (0.95-0.98)	<0.001		
Δ at 6 months	0.96 (0.91-1.01)	0.084		
Δ at 1 year	0.96 (0.92-1.00)	0.035	0.92 (0.87-0.97)	0.001
Six-minute walk test				
Baseline SpO2	-	-		
Nadir SpO2	0.93 (0.90-0.97)	<0.001		
Distance				
baseline	0.99 (0.99-1.00)	<0.001	0.99 (0.98-1.00)	0.005
Δ at 6 months	1.00 (0.98-1.01)	0.557		
Δ at 1 year	1.00 (0.97-1.02)	0.708		
GAP stage				
Stage I	ref			
Stage II	2.95 (1.67-5.22)	<0.001		
Stage III	5.46 (2.83-10.53)	<0.001		

HR, hazard ratio; CI, confidence interval; BMI, body mass index; FVC, forced vital capacity; DLCO, diffusing capacity of the lungs for carbon monoxide; GAP, sex-age physiology. †Home oxygen use during the follow-up period.

Table S3. Receiver operating characteristic analysis of changes in pulmonary function and exercise capacity for predicting mortality during 1-year follow-up period in patients with IPF not receiving antifibrotic therapy

Variable	Cut-off value	AUC (p-value)	Sensitivity, %	Specificity, %	PPV, %	NPV, %
ΔFVC at 12 months	-6.5	.665 (0.133)	62.5	79.4	27.8	94.3
ΔDLCO at 12 months	-2.5	.869 (0.003)	100.0	62.5	22.2	100.0
Δ6MWD at 12 months	24.5	.182 (0.500)	100.0	18.2	10.0	100.0

AUC, area under the curve; FVC, forced vital capacity; DLCO, diffusing capacity of the lungs for carbon monoxide; 6 MWD, six-minute walk distance; PPV, positive predictive value; NPV, negative predictive value

Figure S1. Comparison of pulmonary function and exercise capacity changes according to survival status in patients with IPF not receiving antifibrotics. (a-c) Patients who did not receive antifibrotic treatment. In the non-treatment group, no statistically significant changes over time were observed between the non-survivor and survivor cohorts.

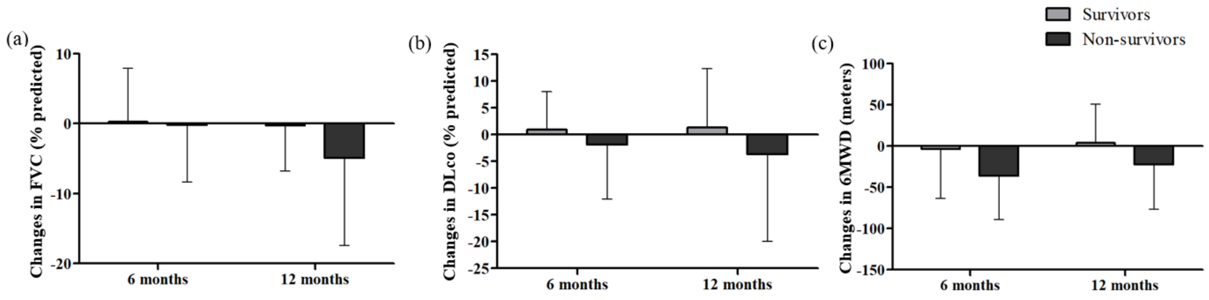

FVC, forced vital capacity; DLCO, diffusing capacity of the lungs for carbon monoxide; 6 MWD, six-minute walk distance.
